# Endoscopic ultrasound-guided colorectal anastomosis using a lumen-apposing metal stent for complete anastomotic stricture

**DOI:** 10.1055/a-2767-1774

**Published:** 2026-01-20

**Authors:** Rafael Prado Pessoa, Caroline Assis Aleixo Chaves, Júlia Gallo de Alvarenga Mafra, Eduardo Seubert Coelho Vieira, Lucas Gallo de Alvarenga Mafra, Luiz Claudio Miranda Rocha, Rodrigo Roda

**Affiliations:** 1 223018 Endoscopy Division, Mater Dei Santo Agostinho, Belo Horizonte, Brazil; 2 219764 Endoscopy Division, Hospital das Clínicas da Universidade Federal de Minas Gerais, Belo Horizonte, Brazil

Lumen-apposing metal stents (LAMSs) were designed for pancreatic fluid collections and endoscopic ultrasound (EUS)-guided biliary drainage, but their applications have expanded to include enteric anastomosis creation and the management of gastrointestinal strictures, owing to their variable diameters, anti-migration design, and relatively simple deployment technique 1 2 3 .

**Fig. 1:**
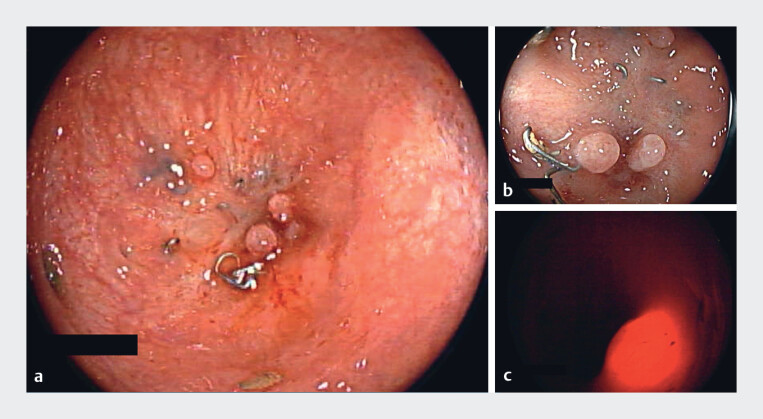
An endoscopic view of the colorectal anastomosis showing the complete stricture. **a** A view from the proximal segment. **b** A view from the rectal side. ( **c** ) Attempted transillumination, without a safe window to allow therapeutic intervention.

A 74-year-old man underwent rectosigmoidectomy with primary anastomosis for recurrent diverticulitis. On postoperative day (POD) 7, he developed an anastomotic leak requiring surgical revision and creation of a loop ileostomy. Seven months later, intestinal continuity was restored. However, on POD 3 after stoma reversal, he developed an acute obstructive abdomen. Urgent flexible sigmoidoscopy revealed a complete colorectal anastomotic stricture. The patient then underwent urgent surgery with the creation of a loop colostomy. Two months later, colonoscopy confirmed persistent complete obstruction ( Fig. 1 ).

**Fig. 2:**
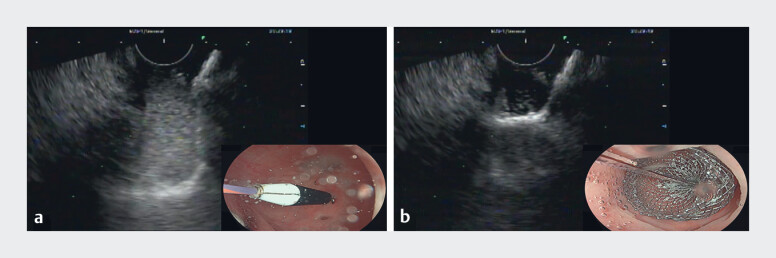
Endosonographic and endoscopic images of LAMS deployment. **a** and **b** Deployment of the LAMS distal flange. LAMS, lumen-apposing metal stent.

EUS-guided colorectal anastomosis with LAMSs was selected for recanalization ( Video 1 ). A colonoscope was advanced through the colostomy while a linear echoendoscope was inserted transrectally. A 19-gauge FNA needle was used for access, followed by guidewire introduction and deployment of a 20 × 10-mm electrocautery-enhanced LAMS (Hot Axios; Boston Scientific, Marlborough, Mass, USA; Fig. 2 ). Contrast-tinged water confirmed immediate communication between the segments.

EUS-guided colorectal anastomosis with a lumen-apposing metal stent for the management of the complete anastomotic stricture. EUS, endoscopic ultrasound.Video 1

**Fig. 3:**
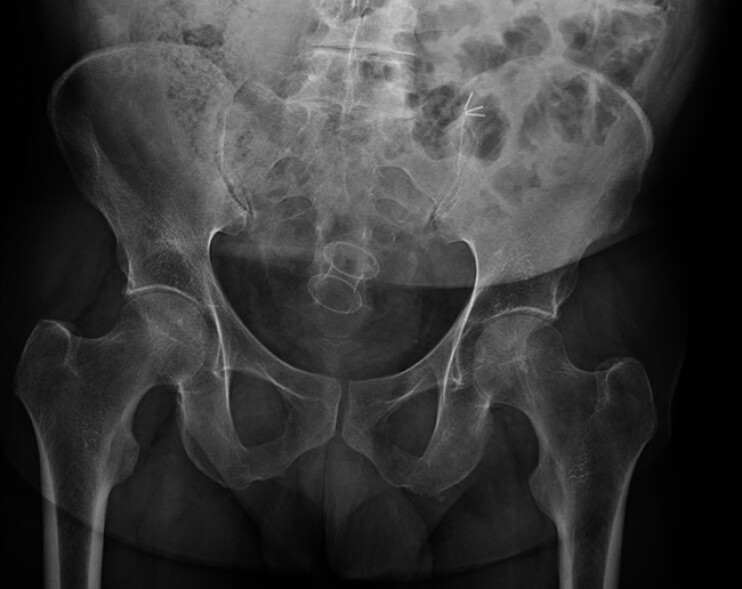
Pelvic radiography 1 week after LAMS placement showing an appropriate position and expansion. LAMS, lumen-apposing metal stent.

**Fig. 4:**
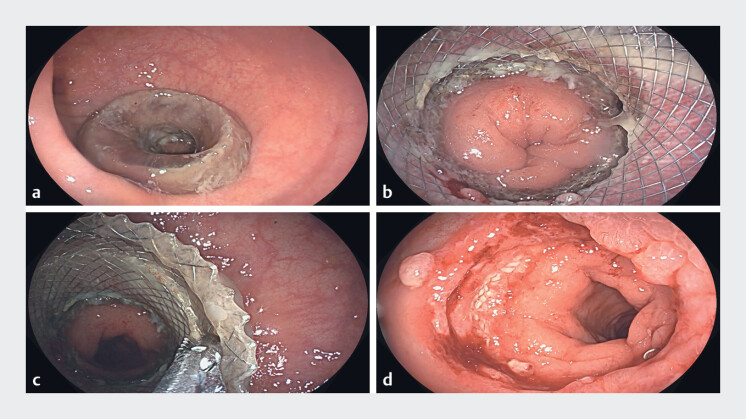
Endoscopic images from colonoscopy performed 4 weeks after LAMS placement. **a** The LAMS in the correct position, covered with the mucus. **b** The LAMS after mucus clearance, with no evidence of complications such as migration, bleeding, or ulceration. **c** LAMS removal using a retrieval forceps, without difficulties. **d** Appearance of the anastomotic site after LAMS removal. LAMS, lumen-apposing metal Stent.

**Fig. 5:**
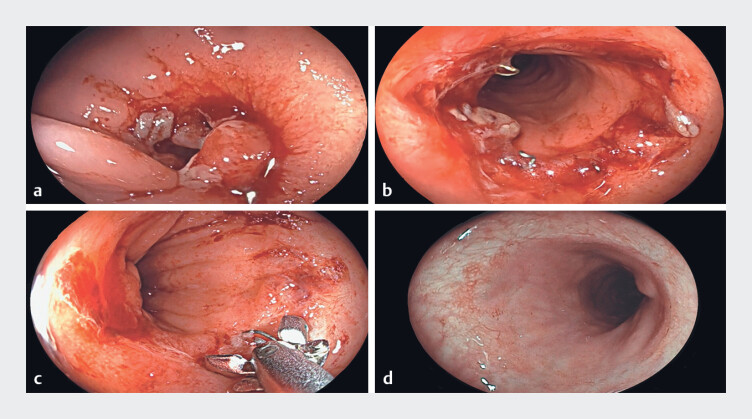
**a** A partial anastomotic stricture visualized 1 week after stent removal, not allowing passage of the 12.8-mm standard colonoscope. **b** An endoscopic appearance after the first dilation session. **c** The surgical clips were removed with forceps during the dilation sessions. **d** An endoscopic appearance on follow-up colonoscopy after four dilation sessions.

A pelvic radiograph 1 week later demonstrated appropriate LAMS expansion ( Fig. 3 ). At 4 weeks, colonoscopy showed a well-positioned stent without adverse events, allowing safe removal and revealing a healed anastomosis ( Fig. 4 ). Persistent strictures required four hydrostatic balloon dilations of up to 20 mm to achieve satisfactory patency. The surgical clips were removed with forceps during the dilation sessions. The patient was subsequently cleared for colostomy takedown ( Fig. 5 ).

EUS-guided colorectal anastomosis using LAMSs proved safe and effective for complete anastomotic obstruction in this case, enabling restoration of intestinal continuity and avoiding additional surgery. Despite promising outcomes, questions remain regarding an ideal dwell time and the need for larger studies 1 2 3 4 5 .

Endoscopy_UCTN_Code_TTT_1AQ_2AF
